# Antiretroviral Treatment Scale-Up and Tuberculosis Mortality in High TB/HIV Burden Countries: An Econometric Analysis

**DOI:** 10.1371/journal.pone.0160481

**Published:** 2016-08-18

**Authors:** Isabel Yan, Eran Bendavid, Eline L. Korenromp

**Affiliations:** 1 Department of Economics and Finance, City University of Hong Kong, Kowloon, Hong Kong; 2 Division of General Medical Disciplines, Stanford University, Stanford, California, United States of America; 3 Center for Health Policy and the Center for Primary Care and Outcomes Research, Stanford University, Stanford, California, United States of America; 4 Department of Public Health, Erasmus MC, University Medical Center, Rotterdam, the Netherlands; 5 Avenir Health, Geneva, Switzerland; Fundacao Oswaldo Cruz, BRAZIL

## Abstract

**Introduction:**

Antiretroviral therapy (ART) reduces mortality in patients with active tuberculosis (TB), but the population-level relationship between ART coverage and TB mortality is untested. We estimated the reduction in population-level TB mortality that can be attributed to increasing ART coverage across 41 high HIV-TB burden countries.

**Methods:**

We compiled TB mortality trends between 1996 and 2011 from two sources: (1) national program-reported TB death notifications, adjusted for annual TB case detection rates, and (2) WHO TB mortality estimates. National coverage with ART, as proportion of HIV-infected people in need, was obtained from UNAIDS. We applied panel linear regressions controlling for HIV prevalence (5-year lagged), coverage of TB interventions (estimated by WHO and UNAIDS), gross domestic product per capita, health spending from domestic sources, urbanization, and country fixed effects.

**Results:**

Models suggest that that increasing ART coverage was followed by reduced TB mortality, across multiple specifications. For death notifications at 2 to 5 years following a given ART scale-up, a 1% increase in ART coverage predicted 0.95% faster mortality rate decline (p = 0.002); resulting in 27% fewer TB deaths in 2011 alone than would have occurred without ART. Based on WHO death estimates, a 1% increase in ART predicted a 1.0% reduced TB death rate (p<0.001), and 31% fewer deaths in 2011. TB mortality was higher at higher HIV prevalence (p<0.001), but not related to coverage of isoniazid preventive therapy, cotrimoxazole preventive therapy, or other covariates.

**Conclusion:**

This econometric analysis supports a substantial impact of ART on population-level TB mortality realized already within the first decade of ART scale-up, that is apparent despite variable-quality mortality data.

## Introduction

The HIV epidemic, and the expansion of antiretroviral therapy (ART), have been dominant drivers of adult mortality in resource-limited countries during the 1990s and 2000s [1–3]. Between 2003 and 2012, the number of people receiving ART increased from 100,000 to over 10 million, mostly in sub-Saharan Africa, and adult mortality declined where ART became available [4–8].

The extent to which ART coverage is related to declining HIV-associated TB is unknown. In some contexts, TB is responsible for more than a fourth of all deaths among HIV-infected individuals, and providing ART has been linked to lower TB mortality [9–14]. Epidemiologic models estimate that expanding ART could halve the number of HIV-related TB deaths [10]; and ART is considered a highly cost-effective component of TB control in sub-Saharan African countries [11, 15, 16].

Models of TB epidemiology have been influential in supporting ongoing funding and prioritization of ART as a key intervention for TB control, despite a dearth of population-level evaluations of the relationship between ART expansion and TB mortality. Epidemiological and statistical models used by international health agencies, which assume population-level benefits inevitably result from increasing intervention coverage or even just service delivery numbers, have been criticized as being over-optimistic, and incapable of accounting for differences between theoretical efficacy and real-world effectiveness. For countries with good national-level mortality statistics and early ART scale-up, such as Thailand [17], South Africa, Botswana, [18] and Brazil [19], predicted mortality declines appear to be stronger than empirical data suggest.

To address this evidence gap, this paper examines the relation between ART expansion and trends in population-level mortality from TB, from empirical data across high HIV/TB burden countries.

## Methods

We test the hypothesis that expansion of antiretroviral therapy (ART) in countries with a high HIV/TB burden was followed by reductions in TB mortality. Using longitudinal country-level data on TB mortality and ART coverage, we test this relationship and its chronology using panel regressions. We analyzed mortality trends in 41 countries identified by the WHO Stop TB program as those with the highest dual burden of HIV and TB [20]. We assembled panel data on TB mortality, ART coverage, and two other key HIV/TB interventions: isoniazid preventive therapy (IPT), which decreases TB disease incidence and hence TB mortality, and cotrimoxazole preventive therapy (CPT), also believed to contribute to reducing mortality from TB in conjunction with its effect of reducing the incidence of other important HIV-associated opportunistic conditions that drive mortality [21–27]. All data used (detailed in S1 Table) were from public sources.

### TB mortality data

We used two alternative metrics of TB mortality: (i) The World Health Organization (WHO) TB department’s modeled estimates of TB deaths from a triangulation of data from national TB and HIV programs, vital registration, and TB surveys; and (ii) our estimates based on reported TB death notifications, which we adjusted using WHO’s “indirect method” [9, 28]. We analyzed both measures in parallel, because each had different advantages and disadvantages.

The WHO Stop TB country mortality estimates are published annually. These are derived from mathematical models that synthesize a variety of sources, including research studies about the natural history and interaction between HIV and TB (from selected settings), country- and year-specific data about patient burdens, intervention coverage and quality from national TB and HIV/AIDS programs, vital registration (where available), and TB prevalence surveys and studies [9, 20, 28, 29]. The resulting estimates are arguably the most balanced ones possible to indicate overall TB mortality, whereas TB death notifications made up only around 15% of the WHO estimates over the period analyzed [9, 30, 31]. However, the WHO models use ART coverage as one predictor of mortality among HIV-infected TB patients [28], raising concerns about circularity when using this source to estimate the effect of ART coverage on TB mortality.

Our “indirect method” is based on published TB death notifications. We summed TB deaths across eight patient categories that National TB Programs (NTPs) report separately: (1) HIV-negative new smear-positive or culture-positive cases; (2) HIV-negative smear-negative cases (including extra-pulmonary TB and unknown smear status); (3) HIV-positive new smear-positive or culture-positive cases; (4) HIV-positive smear-negative cases (including extra-pulmonary TB and unknown smear status); (5) HIV-negative re-treatment cases (comprising the sub-categories of re-treatment, other re-treatment, relapse, treatment after default, and failure after default); (6) HIV-positive re-treatment cases (including the same sub-categories as in (5); (7) cases with multidrug-resistant (MDR) TB; and (8) cases with extensively drug-resistant (XDR) TB. For each category we first estimated the case-fatality rate, as the number of notified deaths in the treatment cohort divided by size of that cohort. We then estimated deaths by multiplying this case fatality rate by the total cases notified in that case category. Finally, since cases notified typically represent only a portion of overall TB cases [30], we divided the sum of notified TB deaths by the case detection rate (for each country-year specifically), i.e. by the ratio between notified cases and total incident cases.

For both methods, the number of TB deaths was normalized by countries’ population size (in 100,000s) for the same year. Data were analyzed for the period 1996 (the first year with reasonably complete data for key variables) through 2011 (the latest year with NTP treatment cohort outcome data available as of 2014).

### ART coverage data

ART coverage estimates, expressed as proportion of people receiving ART among all people living with HIV (PLWH) in need of ART (defined as CD4<350/μL), are estimated annually for each country by UNAIDS [7].

### Covariates

We used several covariates to control for contemporaneous drivers of TB mortality, including national TB control programs. These included:

- Isoniazid Preventive Therapy (IPT) coverage. IPT is recommended for HIV-infected individuals with latent TB [32–34]. We analyzed IPT coverage as the number of people given IPT, as reported by WHO Stop TB, divided by the number of HIV-infected individuals in the same country-year.
- Cotrimoxazole Preventive Therapy (CPT) coverage. CPT is recommended for HIV-infected TB patients [21, 22, 32, 35]. We estimated CPT coverage as the number of notified HIV-infected TB patients on CPT, divided by the number of HIV-infected TB patients estimated by WHO [36].

In addition, analyses included the following time-varying correlates of mortality:

- HIV prevalence among adults (15–49 years), with a 5-year lag to allow for the typical interval from peak in HIV prevalence to peak in HIV-related TB morbidity and mortality [15];
- Percentage of national population living in urban areas, from the World Development Indicators [37];
- Gross Domestic Product per capita, measured in constant (purchasing power) 2005 international dollars [37];
- National health expenditure per capita, which may influence mortality including from TB [38].

In addition to these time-varying covariates, all analyses included country fixed-effects to control for time-invariant differences among countries, including fixed baseline or pre-existing differences, such as in disease burden levels.

### Missing data & imputation

Missing country-years of notifications (3.8% of total country-years analyzed) were imputed by linear extrapolation between the closest years with data. This was done to TB death notifications (3.8% of country-year observations were imputed), CPT coverage (3.3%), and IPT coverage (9.2%). ART coverage data were complete.

For India, new smear-negative TB case notifications had a low outlier in 1997 in the online database, which was however not consistent with written reports [39]; therefore we replaced the 1997 value using simple linear imputation between 1996 and 1998 values. For Chad, no patients were reported as receiving IPT in Chad, and we assumed zero IPT coverage throughout all years. Coverage of CPT, IPT and ART prior to the first year with data was assumed to be 0 for all countries. Graphical plots of the data indicates that this assumption provides a natural backward extrapolation of the ART and IPT data (S1 File). We opted to also set the baseline for CPT coverage at 0, as for ART and IPT, for simplicity, for consistency with the approach taken for ART and IPT, and because the 0 imputation makes our analysis conservative guarding against inflated effect sizes.

Of the 41 high HIV/TB burden countries, Brazil and Sudan lacked a point estimate of the HIV-infected population size; for those countries, we averaged the UNAIDS ‘high’ and ‘low’ estimates. China, Central African Republic, and the Russian Federation had no estimates of HIV prevalence of HIV-infected population, and were excluded from analysis. Zimbabwe was excluded due to lack of GDP data after 2003.

### Statistical Analysis

Relationships between TB mortality, ART coverage, and other relevant covariates were evaluated from 1996 to 2011 for 37 countries. To allow for lags between intervention implementation and mortality effect, the fixed effects panel models lagged ART, CPT and IPT coverage by 1–5 years, and capped the duration of possible mortality effects at three years [40]. For example, with a 1-year lag, ART coverage in 2003 was analyzed for mortality effects over the period from 2004 to 2006. GDP per capita used the same lag as ART, CPT and IPT.

### Sensitivity analyses

Sensitivity analyses examined the robustness of findings against uncertainties in key exposure and outcome variables.

A first analysis repeated the primary specification without GDP and health expenditure covariates, so that Zimbabwe could be included (giving 38 instead of 37 countries analyzed). Second, we repeated the indirect method for smear-positive TB only, instead of all 8 death categories. While smear-positive deaths are less complete (comprising about 57% of the deaths estimated by our indirect method across the 8 categories) and less representative of all TB deaths, the relative clarity in their definition and the long duration of this variable’s availability (since 1995 for all countries) relax concerns over bias and mis-measurement, especially compared with smear-negative deaths (reported only since 2005).

A third sensitivity analysis expanded the analysis to include low-HIV countries, as a type of placebo-based control to check if effects of ART are strongest in, or even limited to, countries with high HIV prevalence–as expected for a true causal effect–and do not merely represent confounding as might occur when improving ART coverage happens to coincide with improving TB program coverage.

Fourth, we used HIV funding to proxy for ART coverage, using HIV disbursements from foreign aid for programs such as the Global Fund to fight AIDS, Tuberculosis and Malaria (Global Fund) and the USA’s PEPFAR, which drove scale-up of ART in low- and middle-income countries since 2002 [41]. The following ‘instrumental’ predictors were used instead of ART coverage:

- Global Fund and PEPFAR HIV disbursements, expressed per HIV-infected population (with Global Fund TB disbursements as covariate);
- Foreign aid for HIV control from all donors, per HIV-infected population [42].

A final sensitivity analysis added the TB case detection rate as time-varying covariate, as an indicator of TB program performance, which in some countries improved in parallel with ART coverage and might thus potentially confound the mortality impact of ART. The TB case detection rate is a sensitive indicator of national TB program performance, with considerable variation across countries and within the time horizon evaluated [20], but it was also used as an intermediate variable in our notification-based TB mortality outcome estimate. To avoid circularity, this analysis was therefore limited to the WHO-estimated TB mortality as the outcome.

The analytic code is Available the authors upon request; all analyses were performed using Stata 12.1 (Statacorp).

## Results

### Intervention scale-up and TB mortality decline–descriptive analysis

**Fig 1:**
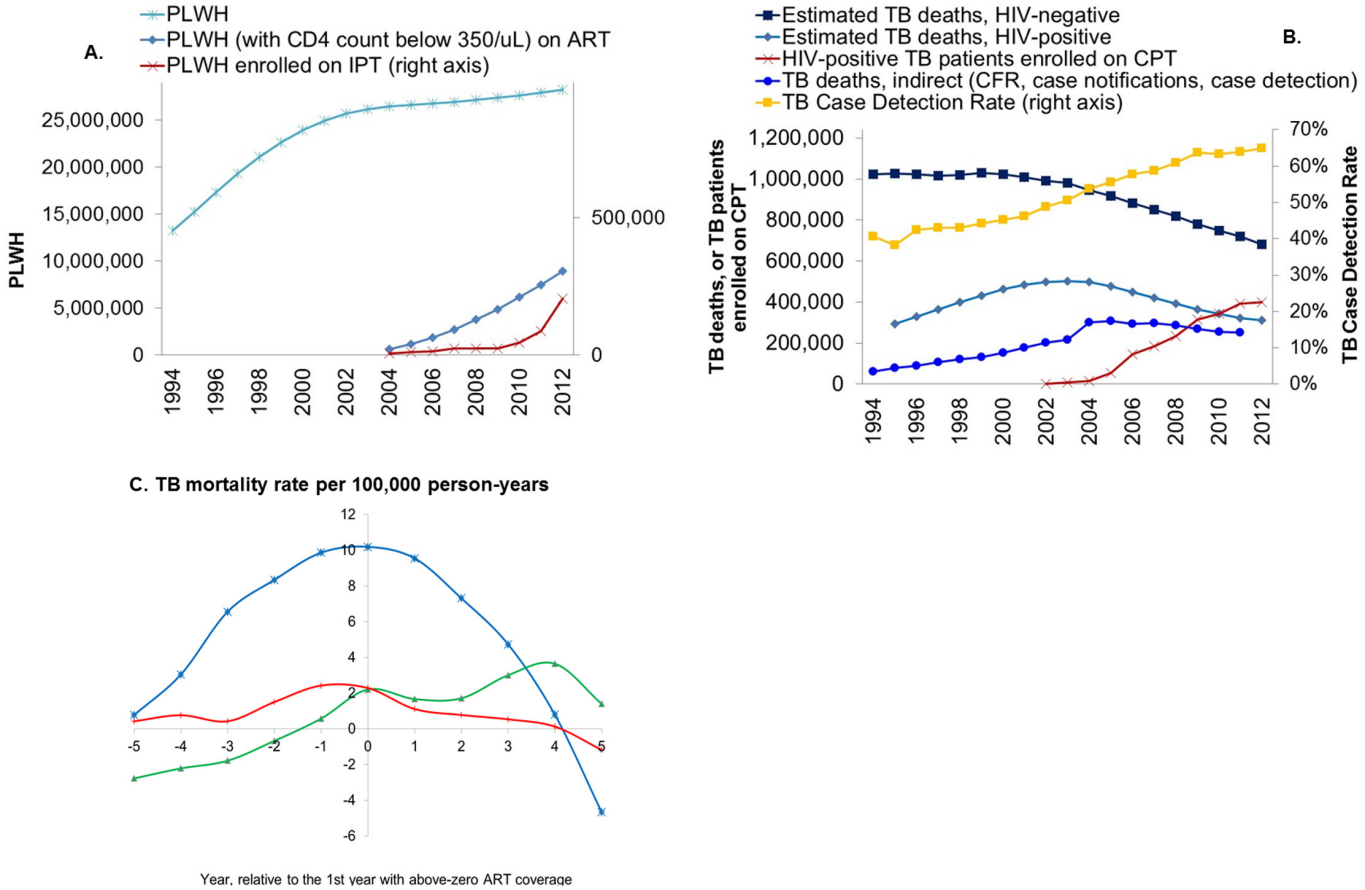
(a) HIV and (b) TB mortality and intervention coverage, and (c) TB mortality relative to start of ART scale-up. *Totals across 41 high TB/HIV burden countries*. *In Fig 1c*, *the mortality rate of each country (which are all weighted equally) is included as the difference between the country’s year-specific mortality (per 100*,*000 person-years) and its mean mortality over 1996 to 2011 (de-meaned)*. *Blue line with ‘x’ marker*: *WHO estimate of TB deaths; Green line with triangle marker*: *Notification-based TB deaths with authors’ adjustment for notification completeness*, *including eight death notification categories (see* Methods*); Red line with ‘+’ marker*: *Notification-based TB deaths with authors’ adjustment for notification completeness*, *limited to smear-positive patients*.

Across 41 high TB/HIV burden countries, ART started around 2004, with a fairly linear scale-up through 2012, reaching about 30% of PLWH in need of ART by 2012 (Fig 1a). Coverage of CPT also scaled-up fairly linearly from 2004, though levelling off by 2011–2012 (Fig 1b). CPT reached 71% of known HIV-infected TB patients–but a much smaller proportion of the total (larger) number of HIV-infected TB patients–by 2012. IPT coverage was scaled-up much more slowly and modestly, mainly from 2009 onwards. IPT notification data are far from complete, but across 30 countries that reported in 2012, 30% of PLWH newly enrolled in HIV care got initiated on IPT. In contrast to the marked acceleration in scale-up of these HIV-related interventions, the TB case detection rate, an indicator of the performance of DOTS programs, continued its preceding gradual increase through 2012 (Fig 1b).

Over the period of HIV-related intervention scale-up from 2004–5, while annual numbers of PLWH continued a gradual (though slowing) increase through 2012 (Fig 1a), annual TB deaths started to decline (Fig 1b). This mortality decline is most apparent in the WHO estimates for HIV-uninfected TB deaths (linear decrease from 2003 to 2012), whereas WHO-estimated TB deaths among HIV-infected people continued to rise until 2004, with a reversal and subsequent decline from 2005.

TB deaths from notifications data (with authors’ adjustment for completeness) rose through 2004, followed by stable annual numbers thereafter. A sudden rise from 2003 to 2004 in part reflects a reporting artefact, of smear-negative TB deaths that were reported from 2004 only–which our adjustment by annual case detection rates did not fully compensate for. In year 2011, notifications with our adjustment captured 19% of WHO-estimated TB deaths (in HIV-infected and HIV-uninfected individuals combined).

### TB mortality decline relative to intervention onset–descriptive analysis

Considering trends in TB mortality relative to the start of ART, IPT and CPT in each country, WHO-estimated declines in TB mortality started around the same time of ART scale-up (Fig 1c, blue line). Notifications-based TB mortality, in contrast, continued to rise after onset of ART, to start a decline only around 5 years after onset of ART (Fig 1c, green line). The delayed decline in part reflects that many NTPs in high HIV/TB-burden countries started to report non-smear-positive cases and deaths only in later years of the evaluation period. When considering only smear-positive subset, notified deaths fluctuated over years before ART start and started a decline around the start of ART scale-up (Fig 1c, red line).

Estimated TB mortality declines preceded the start year of CPT and IPT in most countries (not shown), which typically took off only years after start of ART.

### Panel regressions

In panel regressions, WHO-estimated TB mortality declined around 1% faster in countries and years with ART coverage scale-up (Table 1, top), an effect that was highly significant (p<0.001) when considering TB mortality decline over years 1–3, 2–4 or 3–5 after onset of ART scale-up. Slightly smaller and less significant effects remained apparent when considering TB mortality patterns over a later period after ART scale-up (lags of 4–6 or 5–7 years). In contrast, these statistical models did not reveal any significant independent effect of scale-up of CPT and IPT or health expenditure per capita. Independent of ART coverage, TB mortality decline was larger in countries with higher HIV prevalence (p<0.006 across models).

**Table 1:** Panel regressions of effects of ART coverage on TB death rate, using as outcome variable: (top) WHO-estimated TB deaths as outcome variable; (bottom) authors’ notification-based TB deaths (applying indirect adjustment).

*Assumed minimum lag*, *from start of ART/CPT/IPT to impact on TB mortality*	*1 year lag*	*2 years lag*	*3 years lag*	*4 years lag*	*5 years lag*
**Outcome: WHO-estimated TB deaths:**					
ART coverage	-0.0100** (0.000)*	**-0.0097 (0.000)**	**-0.0097 (0.000)**	**-0.0091 (0.003)**	**-0.0081 (0.050)**
CPT coverage	-0.0005 (0.14)	-0.0005 (0.26)	-0.0006 (0.29)	-0.0007 (0.29)	-0.0012 (0.18)
IPT coverage	**-0.0355 (0.084)**	**-0.0337 (0.040)**	-0.0146 (0.57)	-0.0385 (0.23)	-0.0507 (0.20)
HIV prevalence (lagged 5 years)	**0.0641 (0.000)**	**0.0589 (0.000)**	**0.0556 (0.002)**	**0.0551 (0.004)**	**0.0544 (0.006)**
% of urban population	-0.0141 (0.43)	-0.0198 (0.25)	-0.0308 (0.11)	**-0.0373 (0.070)**	**-0.0427 (0.046)**
GDP per capita (in 2005 USD, PPP-adjusted, natural log)	0.0333 (0.89)	-0.0158 (0.95)	-0.0776 (0.77)	-0.1384 (0.62)	-0.1847 (0.52)
Health expenditure per capita (in 2005 USD, natural log)	-0.0820 (0.22)	-0.1121 (0.14)	-0.1170 (0.20)	-0.1336 (0.20)	-0.1435 (0.20)
Number of countries	37	37	37	37	37
Number of country-year observations	402	402	402	402	402
R-square (within)	69%	67%	64%	61%	59%
**Outcome: notification-based TB deaths:**					
ART coverage	-0.0095** (0.003)*	**-0.0091 (0.006)**	**-0.0088 (0.011)**	**-0.0086 (0.019)**	-0.0073 (0.10)
CPT coverage	-0.0004 (0.45)	-0.0005 (0.45)	-0.0007 (0.34)	-0.0014 (0.13)	**-0.0019 (0.047)**
IPT coverage	-0.0181 (0.38)	-0.0179 (0.43)	-0.0105 (0.71)	-0.0024 (0.94)	-0.0036 (0.93)
HIV prevalence (lagged 5 years)	**0.0463 (0.096)**	0.0458 (0.19)	0.0435 (0.20)	0.0422 (0.20)	0.0426 (0.17)
% of urban population	**0.0463 (0.096)**	0.0417 (0.12)	0.0335 (0.20)	0.0267 (0.28)	0.0222 (0.35)
GDP per capita (in 2005 USD, PPP-adjusted, natural log)	-0.2416 (0.50)	-0.2901 (0.41)	-0.3505 (0.33)	-0.4164 (0.25)	-0.4539 (0.22)
Health expenditure per capita (in 2005 USD, natural log)	-0.0998 (0.35)	-0.1206 (0.28)	-0.1269 (0.29)	-0.1159 (0.34)	-0.1234 (0.32)
Dummy variable with value 1 for country-years where a National TB Program reported deaths among new smear-negative TB patients treated^$^	**0.3557 (0.000)**	**0.3437 (0.000)**	**0.3426 (0.000)**	**0.3354 (0.000)**	**0.3186 (0.000)**
Number of countries	37	37	37	37	37
Number of country-year observations	400	400	400	400	400
R-square (within)	24%	23%	21%	20%	19%

* P-values are calculated based on robust standard errors. Bold font denotes coefficients statistically significant at p-value <0.05.

** Interpretation of coefficients (example: ART, 2-years lagged): A coefficient of -0.0091 indicates that when ART coverage increases by 1% (for example from 20% to 21%), WHO-estimated TB deaths per 100 000 population decreases by 0.91% (for example, from 200/100 000 to 198.18/100 000).

^$^ The year that NTPs started to report deaths among smear-negative patients varies among the countries (modus 2004). This dummy was included to adjust for confounding between intervention scale-up and the reporting artefact of increases in TB death reporting after onset of NTP’s reporting of smear-negative TB deaths.

Abbreviations: ART = antiretroviral therapy; CPT = Cotrimoxazole Preventive Therapy; GDP = Gross Domestic Product; IPT = Isoniazid Preventive Therapy; GF = Global Fund; PLWH = person living with HIV/AIDS; PPP = purchase power parity; USD = United States Dollars.

With notification-based TB deaths as outcome measure, similar patterns are apparent (Table 1, bottom), although these models fitted less well (R-squares, c.q. proportions of explained variation in mortality, of 19–24%, compared to 59–69% for WHO-estimated mortality). ART coverage scale-up predicts a faster TB mortality decline, again with a near-1% larger mortality decline per percentage ART coverage increase. The effect is most significant (p<0.001) when considering mortality decline starting 1–2 years after ART coverage scale-up, with similar but less significant effects for longer time lags. The effect of ART coverage holds independent from a concurrent effect of NTP notification completeness in increasing notified TB deaths, whereby the start year of reporting of deaths among smear-negative TB patients (analyzed as a dummy variable) marks a strong increase in total notification-based TB deaths.

### Effect of ART scale-up on TB mortality

**Fig 2:**
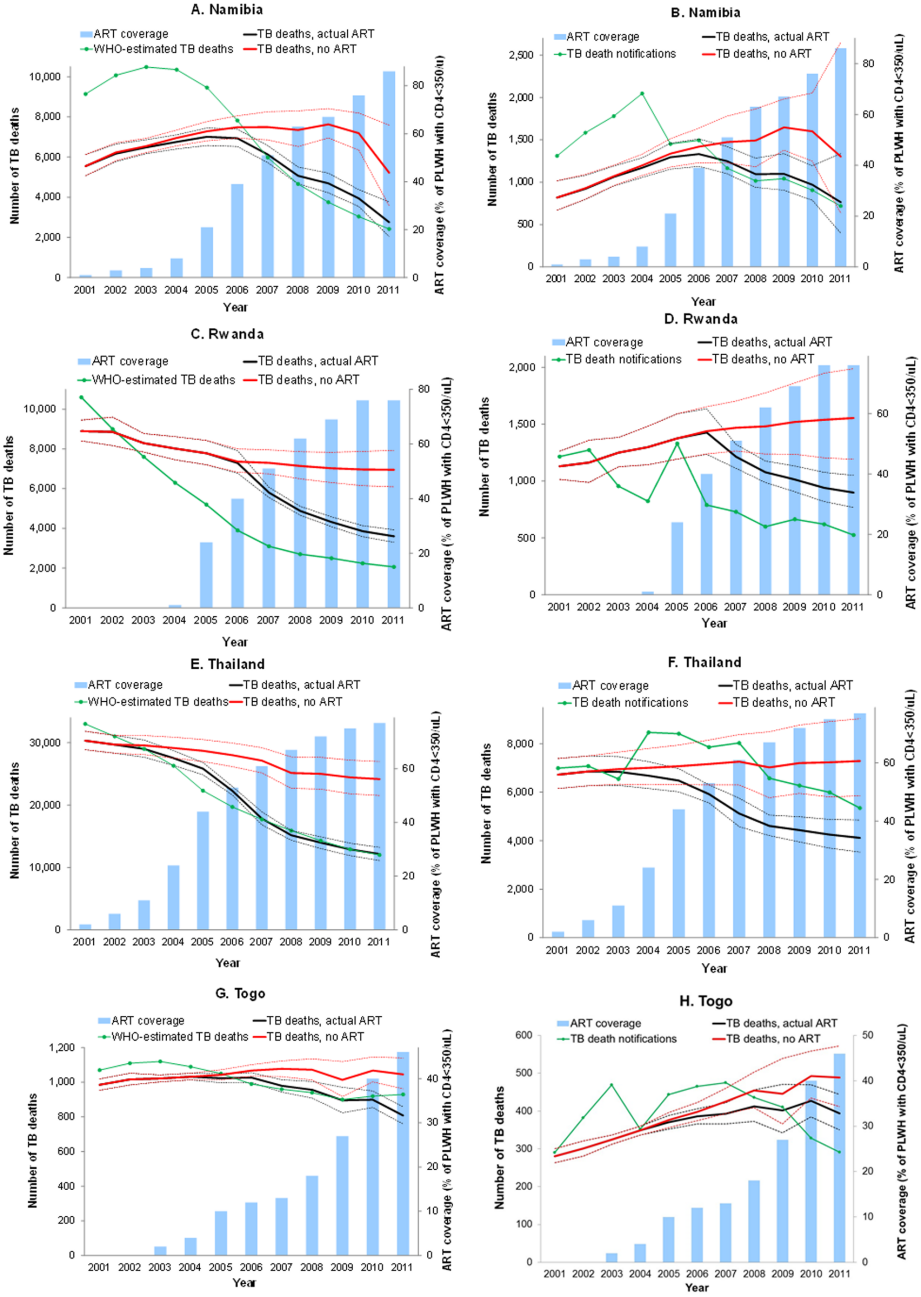
WHO-estimated (left), notification-based (right) and model-predicted TB mortality following ART scale-up, in Namibia; Rwanda; Thailand; and Togo. *Dashed lines give 95% confidence interval. Model predictions use a 2-year time lag*.

Using our regression models to predict the impact of ART scale-up, against a counterfactual of constant zero ART coverage, we find a clear relationship between ART scale-up and subsequent TB mortality declines in countries with considerable ART coverage, as shown in Fig 2 for Namibia, Rwanda and Thailand (Fig 2a–2f). Fig 2 also shows more modest TB declines in Togo, where ART scale-up occurred later and more modestly. For most countries, the WHO-estimated TB death trend is closely matched by our regression model estimate (Fig 2a, 2c, 2e and 2g); and the regression-based counterfactual illustrates the considerable extent to which ART contributed to recent mortality declines.

Notification-based TB death trends are less closely predicted by panel regressions (Fig 2b, 2d, 2f and 2h), reflecting the lesser fit (R^2^) of those models (Table 1) to capture these more error-prone and fluctuating mortality trends. Nevertheless also here the marked observed mortality decline coinciding with start of ART scale-up is reasonably predicted by the regression–whereas in countries with slower ART scale-up such as Togo (Fig 2g and 2h), TB mortality reversal is less prominent, and ART may rather just have slowed and halted an ongoing TB mortality rise, without yet having turned this effect into a net decline.

**Fig 3:**
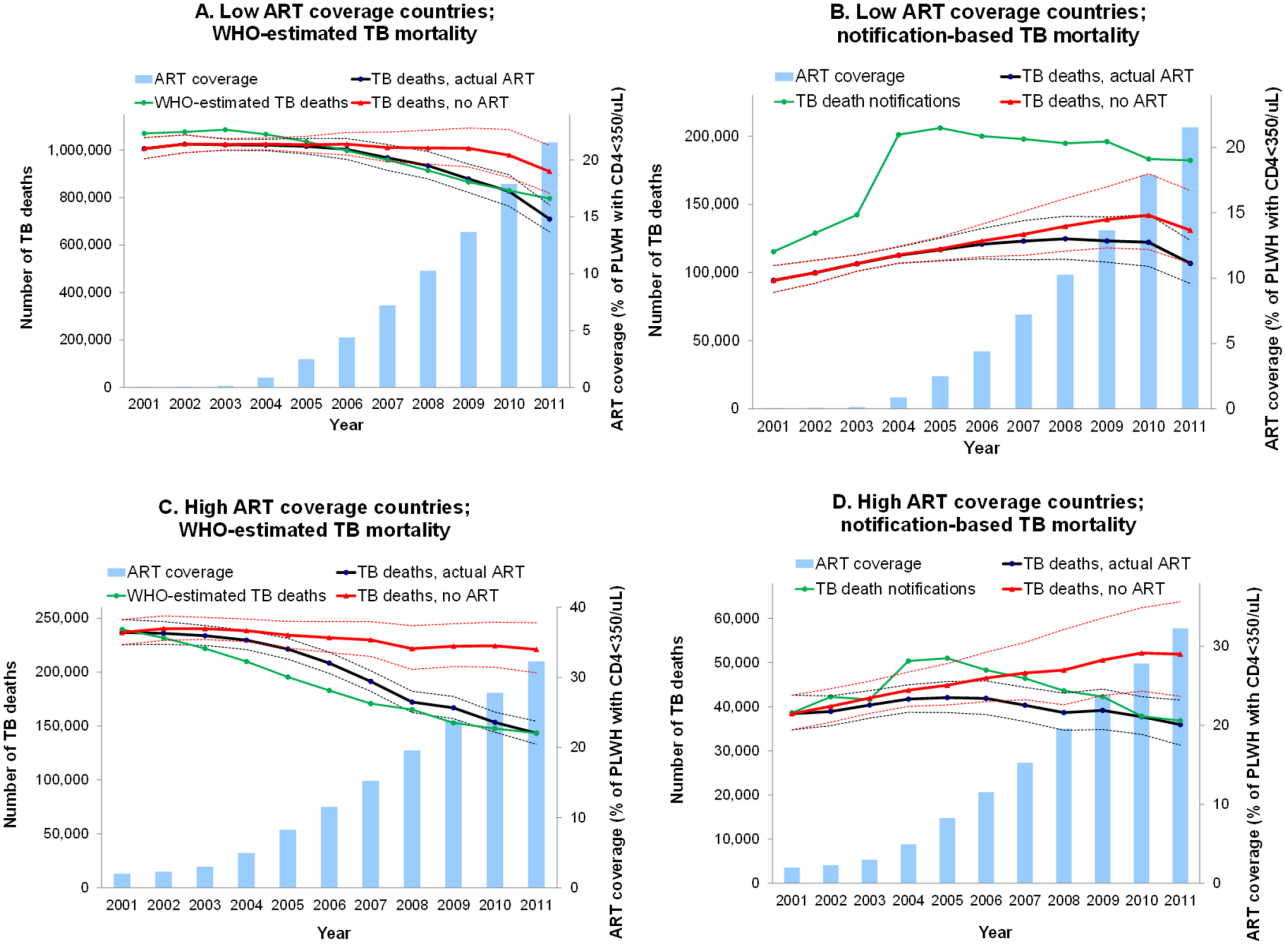
WHO-estimated, notification-based and model-predicted TB mortality, following ART scale-up, in (a & b) 19 high TB/HIV countries with *below* median ART coverage; (c & d) 18 high TB/HIV countries with *above* median ART coverage. *Dashed lines give 95% confidence interval. Model predictions use a 2-year time lag. Split into below median versus above median ART coverage was based on ART coverage averaged over 1996–2012*.

When aggregating the high HIV/TB burden countries into two groups with below-median versus above-median ART coverage, the regressions again illustrate that ART has served to halt, stabilize and started to reverse a preceding rise in TB mortality, into an accelerating mortality decline. This decline is most prominent and earliest in high-ART countries (Fig 3c and 3d), but still also clear in high TB/HIV burden countries with lower ART coverage (Fig 3a and 3b), which by 2011 had reached an overall ART coverage of 22% (compared to 32% among high-ART countries). The ART-associated mortality decline is slightly more prominent in WHO-estimated mortality trends than in notification-based mortality trends. This has two possible explanations: First, WHO-estimated mortality is probably adjusted more completely than our notification-based metric for increasing notification completeness, which likely confounded and partially obscured reductions in population-level mortality. Second, it is possible that WHO assumed over-optimistic effectiveness of ART.

Across the 37 countries modelled, based on notifications there were 143,000 TB deaths in 2011, compared to 197,000 deaths in a counterfactual without ART scale-up, a 27% reduction. Based on WHO estimates, there were 855,000 TB deaths in 2011, compared to 1.23 million deaths in a counterfactual without ART scale-up, a 31% reduction.

### Sensitivity Analyses

Sensitivity analysis generally confirm the robustness of estimated ART effects: in all sensitivity analyses the effect of ART remained significant under either a 2-year lag, or a 4-year lag, or both (Table 2).

**Table 2:** Sensitivity analyses.

*Sensitivity analysis*	*Lag*, *funding to impact*	*ART coverage*: *coefficient (p-value)*	*R-square (within)*	*ART coverage*: *coefficient (p-value)*	*R-square (within)*
*With outcome measure*:		*WHO-estimated TB deaths*		*Notification-based TB deaths (with authors’ adjustment)*	
**Default**	**2 years**	**-0.0097 (0.000)**	**67%**	**-0.0091 (0.006)**	**23%**
	**4 years**	**-0.0091 (0.003)**	**61%**	**-0.0086 (0.019)**	**20%**
Drop GDP and health expenditures per capita, and add Zimbabwe to country dataset	2 years	-0.0105 (0.002)	65%	-0.0086 (0.009)	12%
	4 years	-0.0108 (0.000)	58%	0.0092 (0.028)	9.9%
For notification-based TB deaths (with authors’ adjustment), consider smear-positives only	2 years	n/a	n/a	-0.0085 (0.001)	31%
	4 years	n/a	n/a	-0.0069 (0.036)	29%
Expand the country dataset beyond the 37 high HIV/TB countries with complete predictor data, to 92 countries	2 years	-0.0030 (0.030)	40%	-0.0034 (0.070)	3.5%
	4 years	-0.0013 (0.31)	39%	-0.0028 (0.14)	3.6%
Replace predictor variable ART coverage by HIV and TB disbursements from Global Fund, and PEPFAR disbursements, per PLWH	2 years	GF_HIV+PEPFAR: -0.0004 (0.010) GF_TB: 0.0012 (0.50)	58%	GF_HIV+PEPFAR: -0.0005 (0.000) GF_TB: -0.0007 (0.70)	16%
	4 years	GF_HIV+PEPFAR: -0.0005 (0.055) GF_TB: 0.0011 (0.69)	56%	GF_HIV+PEPFAR: -0.0006 (0.003) GF_TB: -0.0011 (0.59)	13%
Replace predictor variable ART coverage by HIV and TB disbursements from all donors, per PLWH	2 years	HIV disbursement: -0.0003 (0.009) TB disbursement: 0.0012 (0.093)	58%	HIV disbursement: -0.0005 (0.000) TB disbursement: 0.0002 (0.71)	16%
	4 years	HIV disbursement: -0.0003 (0.015) TB disbursement: 0.0005 (0.54)	56%	HIV disbursement: -0.0005 (0.000) TB disbursement: -0.0001 (0.95)	14%
Add TB case detection rate (an indicator of NTP program performance) as predictor variable	2 years	-0.0084 (0.000)	80%	n/a	n/a
	4 years	-0.0080 (0.003)	75%	n/a	n/a

Denotation of p-values and coefficients as in Table 1.

Abbreviations: ART = antiretroviral therapy; GF = Global Fund; GDP = Gross Domestic Product; n/a = not applicable; PLWH = person living with HIV/AIDS; y = years.

When limiting notifications-based deaths to smear-positives, or adding Zimbabwe as additional country in a model dropping economic covariates, the effect of ART remained significant and of equal strength, for both 2-year and 4-year time lags.

Expanding the country dataset beyond high-HIV/TB burden countries to 92 countries reduced overall model fit; but an effect of ART, albeit weaker, remained significant with a 2-year time lag.

When replacing predictor variable ART coverage by HIV and TB funding, HIV funding (from either the Global Fund and PEPFAR combined, or from all donors) significantly predicted faster TB mortality decline, while TB funding (from Global Fund or all donors) had no significant effect.

Finally, adding the TB case detection rate (an indicator of TB program performance) as a covariate did not appreciably change the significant effect of ART in reducing WHO-estimated mortality rates–while an increasing case detection rate independently predicted accelerated TB mortality decline.

## Discussion

Our analysis of the temporal relationship between ART coverage roll-out and TB mortality among high HIV/TB burden countries suggests that ART coverage has markedly contributed to reducing TB mortality, within a few years of the onset of ART roll-out, and independent of any effects of concurrent improvements in national TB program performance.

Assuming a minimum 2-year lag between ART scale-up and start of TB mortality impact, a 1% increased ART coverage predicts a 0.9% larger decline in the notification-based TB death rate (p = 0.002), or a 1.1% reduced TB death rate as estimated by WHO (p<0.001). The similarity of estimated ART effect sizes, between panel regressions predicting WHO-estimated mortality and those predicting notification-based mortality, suggest that the magnitude of ART effectiveness assumed by WHO and its epidemiological advisors [43] is reasonable, and it supports the global target inspired by those models to halve TB-associated HIV deaths by 2015 (compared to 2004) by expanding ART alongside dedicated TB control measures [10]. Nevertheless, our statistical analyses by themselves cannot conclusively establish the size of this causal effect. The role of ART in reducing TB mortality deserves ongoing monitoring and evaluation, over time and geography, as ART gets scaled-up through national programs.

Our findings are consistent with other recent cross-country econometric analyses, whereby funding for HIV control, through the Global Fund among others, predicted accelerated declines in all-cause adult mortality [3, 44, 45]. This is apparently a recent effect, as earlier studies found no effect of PEPFAR funding on TB or TB/HIV mortality [46], or of ART coverage on TB/HIV mortality as estimated by WHO [47].

In contrast to expectation, we failed to find any consistent effects of concurrent CPT or IPT scale-up on reducing population-level TB mortality up to 2011. Randomized trials have found reduced mortality in PLWH on ART thanks to concurrent CPT [26, 27], although the mortality impact has not been established specifically for the sub-group of TB/HIV co-infected patients. This empirical evidence underpinned the WHO recommendation of 2014 for universal CPT and IPT for all PLWH, before and concurrent with ART. The absence of population-level mortality impacts from CPT in our analysis (whether independent from, or in conjunction/synergy with ART) may reflect its initially limited scale-up among TB patients nation-wide across the 41 high TB/HIV burden countries. For IPT, in addition, its less than complete, time-limited effectiveness in preventing TB in high TB/HIV burden settings [26], and the lag time from (prevented) TB infection and ensuing TB activation, disease and TB death, likely explain the absence of country-wide impacts up to 2011.

### Limitations

A key limitation of our study is the quality and completeness, of TB mortality data, and the complexity and uncertainty in TB mortality estimates. Completeness and quality of routine TB death notifications varies among countries and years [30, 31, 48]. While many countries started disaggregating TB mortality by HIV status following the introduction of routine HIV screening at TB treatment enrolment, for most country-years that we analyzed the available data did not distinguish deaths by HIV status. Our indirect method, adjusting notified TB deaths for coverage and case detection rate of DOTS programs and their cohort-based treatment outcome reporting, captured only 19% of WHO-estimated TB deaths in 2011, and this metric was likely biased towards a later TB mortality peak year due to less complete reporting on smear-negative TB cases, although we alleviated this bias using dummy variables indicating the start years of reporting smear-negative deaths. Notably, ART coverage is not used in estimating case detection rates [29], leaving our estimates using TB death notifications insensitive to bias from confounders related to ART coverage. Our complementary outcome measure, model-based estimates from the WHO, did not suffer these biases, but itself included ART coverage as a predictor of mortality, thus creating possible circularity in our estimations.

Despite these limitations, the consistency of findings across both outcome measures supports their robustness. The clear and strong association between ART coverage and TB deaths rates challenge earlier studies that questioned the role of HIV in driving the rise in tuberculosis disease burden, and support assumptions about the clear benefits of ART for TB control used in global epidemiological models. These results underscore the WHO recommendation, adopted in 2013, to expand ART by enrolling PLWH from CD4 count below 500/uL (instead of the former 350/uL) [49], and to further research the potential impact and feasibility of immediate ART (without CD4 threshold) as future policy, toward universal access and the UNAIDS Fast Track Target of reducing HIV incidence and mortality over 2010–2030 by 90% and 80%, respectively, by diagnosing 90% of PLWH, enrolling 90% of known PLWH on ART, and retaining on ART and virally suppressed 90% of those, each from 2020 onward [50].

Future empirical evaluation of TB mortality trends and drivers should hopefully benefit from improved tuberculosis case detection and notification [51–53]. Our ART impact estimates can meanwhile inform cost-effectiveness and cost-benefit assessments about optimal resource allocation between TB and HIV/AIDS prevention and treatment, and other major health burdens and interventions.

## Conclusion

This econometric analysis supports evidence of a substantial beneficial impact of ART in reducing population-level TB mortality. Variations among countries in quality and completeness of TB death notifications and estimates, however, justify rigorous evaluations of TB mortality trends and its drivers, including the effects of large-scale ART programs, and of strategies to further improve ART effectiveness.

## Supporting Information

S1 Table Key TB data used to model drivers of TB mortality trends, for 41 high TB/HIV burden countries. (DOCX)
