# Supplementary material for: Antiretroviral Treatment Scale-Up and Tuberculosis Mortality in High TB/HIV Burden Countries: An Econometric Analysis
Source: PLoS One. 2016 Aug 18;11(8):e0160481. doi: 10.1371/journal.pone.0160481 (PMC4990253; doi:10.1371/journal.pone.0160481)
Supplement: S1 Table — (DOCX) [file pone.0160481.s001.docx]

**S1 Table. Key TB data used to model drivers of TB mortality trends, for 41 high TB/HIV burden countries**

| **Country** | **Notification-based TB mortality rate** | | **WHO-estimated TB mortality rate** | | | **ART coverage %** | | **CPT coverage %** | | | **IPT coverage %** | | | **HIV prevalence %** | | |
| --- | --- | --- | --- | --- | --- | --- | --- | --- | --- | --- | --- | --- | --- | --- | --- | --- |
|  | **2004** | **2011** | **2004** | **2011** | | **2004** | **2011** | **2004** | **2011** | **2004** | | **2011** | | **2004** | **2011** | |
| Angola | 6.22 | 14.79 | 30.71 | 38.65 | | 1 | 35 | 0 | 43 | | 0 | | 0 | 1.9 | | 2.1 |
| Botswana | 23.78 | 31.02 | 194.05 | 84.56 | | 33 | 85 | 0 | 79 | | 3.93 | | 0.23 | 26.2 | | 23.7 |
| Brazil | 2.63 | 2.69 | 4.78 | 4.11 | | 52 | 66 | 0 | 0 | | 0 | | 0 | 0.4 | | 0.3 |
| Burkina Faso | 6.20 | 4.85 | 20.86 | 12.63 | | 6 | 50 | 100 | 98 | | 0 | | 0 | 1.7 | | 1.2 |
| Burundi | 4.88 | 6.08 | 61.77 | 31.45 | | 9 | 47 | 0 | 95 | | 0 | | 0.34 | 2.5 | | 1.4 |
| Cambodia | 18.38 | 7.20 | 120.54 | 72.44 | | 12 | 69 | 0 | 65 | | 0.03 | | 0.61 | 0.9 | | 0.6 |
| Cameroon | 12.66 | 16.43 | 107.51 | 71.37 | | 8 | 35 | 0 | 81 | | 0 | | 0.17 | 5.2 | | 4.7 |
| Central African Republic | 17.67 | 18.32 | 328.21 | 128.49 | | 1 | 20 | 0 | 0 | |  | |  | 6.9 | | 4.9 |
| Chad | 4.32 | 4.16 | 40.94 | 30.63 | | 1 | 33 | 0 | 53 | | 0 | | 0 | 3.5 | | 3.2 |
| China | 1.72 | 0.84 | 6.31 | 3.95 | | 6 | 25 | 0 | 0 | |  | |  | 0 | | 0 |
| Congo | 1.47 | 3.07 | 81.85 | 68.63 | | 1 | 35 | 0 | 3 | | 0 | | 0 | 3.5 | | 3.3 |
| Congo (Democratic Republic) | 14.25 | 10.92 | 66.08 | 61.63 | | 2 | 21 | 67 | 24 | | 0 | | 0 | 0.0 | | 0.0 |
| Côte d'Ivoire | 12.59 | 12.13 | 97.73 | 39.71 | | 3 | 37 | 56 | 80 | | 0 | | 0 | 5.0 | | 3.2 |
| Djibouti | 6.41 | 3.26 | 109.45 | 100.40 | | 3 | 20 | 14 | 0 | | 0 | | 0 | 2.3 | | 1.5 |
| Ethiopia | 8.20 | 5.09 | 65.65 | 29.09 | | 1 | 41 | 0 | 69 | | 0 | | 0.80 | 2.9 | | 1.6 |
| Ghana | 10.20 | 7.29 | 31.80 | 14.02 | | 2 | 36 | 100 | 77 | | 0 | | 0 | 2.1 | | 1.5 |
| Haiti | 17.05 | 11.23 | 66.95 | 41.86 | | 4 | 43 | 0 | 13 | | 0 | | 2.81 | 2.3 | | 1.9 |
| India | 8.70 | 7.86 | 43.30 | 29.97 | | 1 | 36 | 0 | 90 | | 0 | | 0 | 0 | | 0 |
| Indonesia | 5.40 | 3.99 | 40.32 | 28.18 | | 7 | 15 | 0 | 63 | | 0 | | 0 | 0.1 | | 0.2 |
| Kenya | 19.98 | 10.68 | 63.43 | 41.64 | | 4 | 54 | 0 | 100 | | 0 | | 0 | 7.2 | | 6.2 |
| Lesotho | 35.16 | 57.14 | 84.64 | 72.43 | | 2 | 52 | 0 | 96 | | 0 | | 0 | 22.9 | | 23.2 |
| Malawi | 56.23 | 16.79 | 126.89 | 48.52 | | 3 | 47 | 97 | 94 | | 0.03 | | 0.62 | 13.4 | | 10.4 |
| Mali | 6.41 | 3.97 | 15.99 | 11.72 | | 8 | 47 | 0 | 75 | | 0 | | 0 | 1.4 | | 1.1 |
| Mozambique | 40.48 | 20.43 | 218.94 | 223.75 | | 2 | 36 | 0 | 97 | | 0 | | 0.89 | 11.0 | | 11.3 |
| Myanmar | 18.45 | 17.22 | 95.45 | 61.13 | | 1 | 28 | 0 | 100 | | 0 | | 0.25 | 0.8 | | 0.7 |
| Namibia | 100.98 | 40.87 | 510.60 | 137.08 | 8 | | 76 | 33 | 93 | | 0 | | 6.63 | 15.6 | | 13.6 |
| Nigeria | 7.74 | 6.96 | 62.33 | 34.72 | 5 | | 25 | 0 | 59 | | 0 | | 0.05 | 3.7 | | 3.7 |
| Russian Federation | 12.57 | 10.95 | 23.40 | 17.49 | 1 | | 26 | 0 | 0 | |  | |  | 0.8 | | 1.1 |
| Rwanda | 8.74 | 5.56 | 66.81 | 20.10 | 1 | | 76 | 0 | 97 | | 0.30 | | 0 | 3.4 | | 3.0 |
| Sierra Leone | 18.20 | 21.00 | 119.14 | 177.31 | 0 | | 26 | 0 | 6 | | 0 | | 0 | 1.3 | | 1.6 |
| South Africa | 60.12 | 40.38 | 262.72 | 227.33 | 3 | | 33 | 100 | 74 | | 0 | | 2.49 | 17.2 | | 17.3 |
| Sudan | 3.98 | 2.28 | 36.09 | 33.76 | 0 | | 8 | 5 | 0 | | 0 | | 0 | 0.4 | | 0.4 |
| Swaziland | 38.19 | 109.64 | 312.32 | 367.94 | 10 | | 67 | 0 | 93 | | 0 | | 1.02 | 24.8 | | 25.9 |
| Tanzania (United Republic) | 19.67 | 8.35 | 44.04 | 28.91 | 1 | | 36 | 0 | 92 | | 0 | | 0 | 6.4 | | 5.8 |
| Thailand | 12.92 | 9.01 | 40.12 | 19.38 | 24 | | 75 | 0 | 71 | | 0 | | 0 | 1.5 | | 1.2 |
| Togo | 6.35 | 5.07 | 19.67 | 14.21 | 4 | | 40 | 0 | 72 | | 0 | | 0 | 4.2 | | 3.5 |
| Uganda | 11.69 | 10.74 | 113.14 | 49.79 | 13 | | 44 | 0 | 90 | | 0 | | 0 | 6.4 | | 7.0 |
| Ukraine | 6.86 | 10.02 | 25.69 | 19.47 | 2 | | 22 | 0 | 62 | | 0 | | 2.03 | 0.9 | | 0.8 |
| Vietnam | 5.52 | 4.42 | 31.80 | 24.25 | 1 | | 45 | 0 | 62 | | 0 | | 0.52 | 0.4 | | 0.5 |
| Zambia | 22.89 | 7.36 | 116.83 | 85.08 | 9 | | 64 | 0 | 75 | | 0.02 | | 0 | 14.2 | | 12.7 |
| Zimbabwe | 61.83 | 48.76 | 306.83 | 209.60 | 2 | | 59 | 0 | 88 | | 0 | | 0.01 | 20.7 | | 15.2 |

Notes:

- Notification-based TB mortality rate: Our estimates based on reported TB death notifications, which we adjusted using WHO’s “indirect method”. Our “indirect method” is based on published TB death notifications. We summed TB deaths across eight patient categories that National TB Programs (NTPs) report separately: (1) HIV-negative new smear-positive or culture-positive cases; (2) HIV-negative smear-negative cases (including extra-pulmonary TB and unknown smear status); (3) HIV-positive new smear-positive or culture-positive cases; (4) HIV-positive smear-negative cases (including extra-pulmonary TB and unknown smear status); (5) HIV-negative re-treatment cases (comprising the sub-categories of re-treatment, other re-treatment, relapse, treatment after default, and failure after default); (6) HIV-positive re-treatment cases (including the same sub-categories as in (5); (7) cases with multidrug-resistant (MDR) TB; and (8) cases with extensively drug-resistant (XDR) TB. For each category we first estimated the case-fatality rate, as the number of notified deaths in the treatment cohort divided by size of that cohort. We then estimated deaths by multiplying this case fatality rate by the total cases notified in that case category. Finally, since cases notified typically represent only a portion of overall TB cases [[1](#_ENREF_1)], we divided the sum of notified TB deaths by the case detection rate (for each country-year specifically), i.e. by the ratio between notified cases and total incident cases. The number of TB deaths was normalized by countries’ population size (in 100,000s) for the same year.
- **WHO-estimated TB mortality rate**: The WHO TB department’s modeled estimates of TB deaths from a triangulation of data from national TB and HIV programs, vital registration, and TB surveys. The number of TB deaths was normalized by countries’ population size (in 100,000s) for the same year.
- **ART coverage**: ART coverage estimates, expressed as proportion of people receiving ART among all people living with HIV (PLWH) in need of ART (defined as CD4<350/µL).
- **CPT coverage**: Cotrimoxazole Preventive Therapy (CPT) coverage. CPT is recommended for HIV-infected TB patient. We estimated CPT coverage as the number of notified HIV-infected TB patients on CPT, divided by the number of HIV-infected TB patients estimated by WHO.
- **IPT coverage**: Isoniazid Preventive Therapy (IPT) coverage. IPT is recommended for HIV-infected individuals with latent TB [[2-4](#_ENREF_2)]. We analyzed IPT coverage as the number of people given IPT, reported by WHO Stop TB, divided by the number of HIV-infected individuals in the same country-year.
- **HIV prevalence**: HIV prevalence, as % of adults (15-49 years).

**ART coverage data, and imputed 0 values, across 41 countries analyzed:**


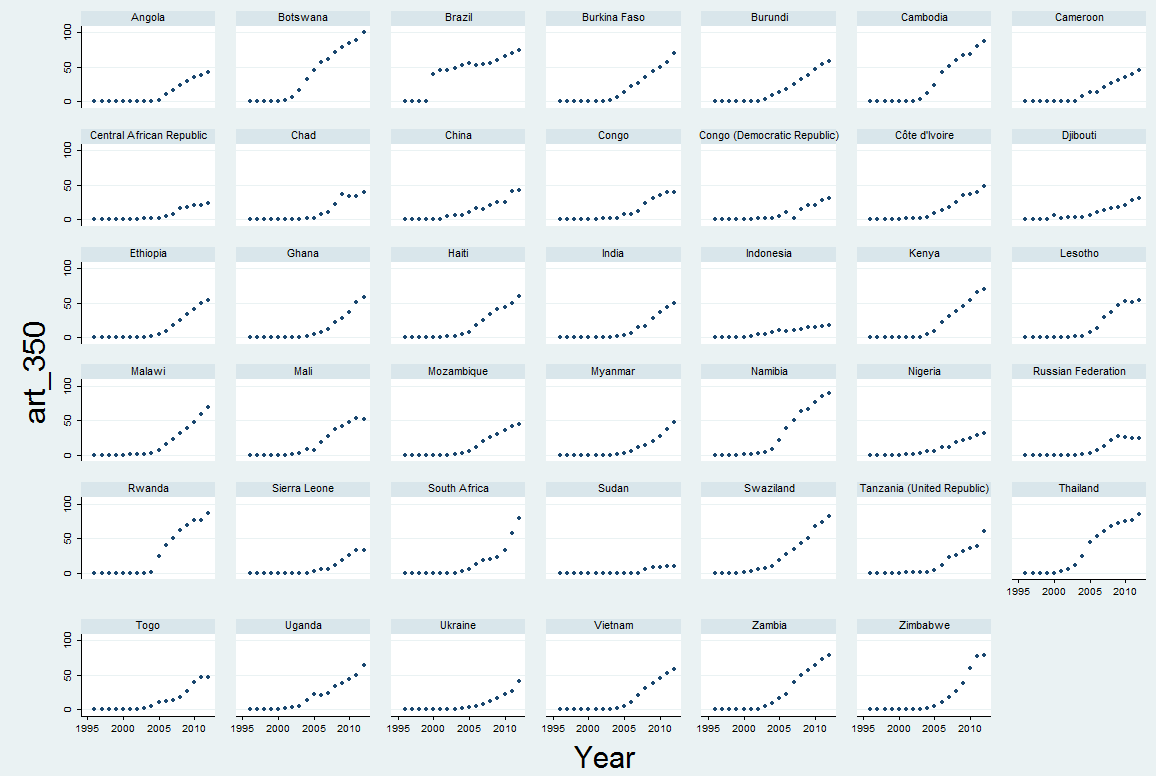


**IPT coverage data, and imputed 0 values, across 41 countries analyzed:**


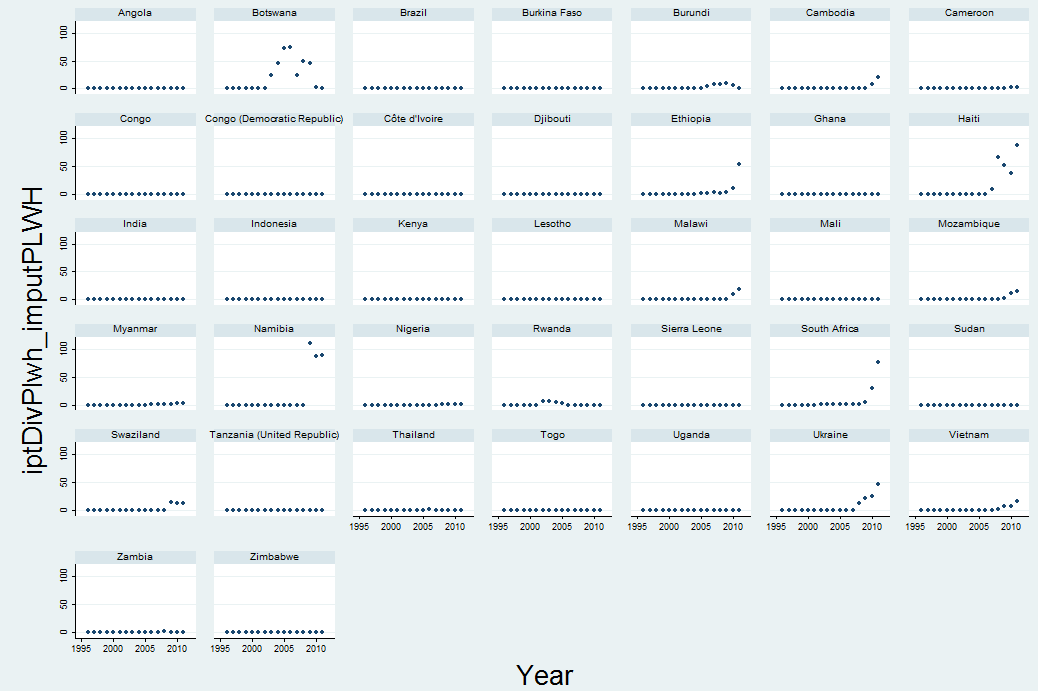


**CPT coverage data, and imputed 0 values, across 41 countries analyzed:**


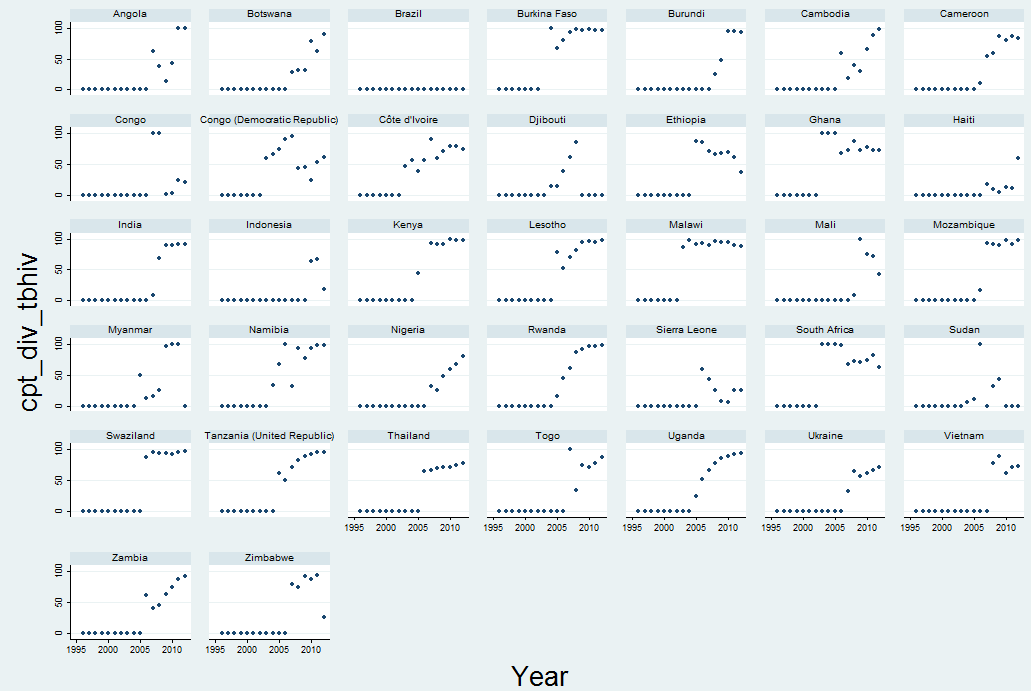


**References for S1 Table**

1. Korenromp EL, Bierrenbach AL, Williams BG, Dye C (2009) The measurement and estimation of tuberculosis mortality. *Int J Tuberc Lung Dis* **13**: 3, 283-303.

2. Uyei J, Coetzee D, Macinko J, Guttmacher S (2011) Integrated delivery of HIV and tuberculosis services in sub-Saharan Africa: a systematic review. *Lancet Infect Dis* **11**: 11, 855-867.

3. Samandari T, Agizew TB, Nyirenda S, Tedla Z, Sibanda T, et al. (2011) 6-month versus 36-month isoniazid preventive treatment for tuberculosis in adults with HIV infection in Botswana: a randomised, double-blind, placebo-controlled trial. *Lancet* **377**: 9777, 1588-1598.

4. Durovni B, Saraceni V, Moulton LH, Pacheco AG, Cavalcante SC, et al. (2013) Effect of improved tuberculosis screening and isoniazid preventive therapy on incidence of tuberculosis and death in patients with HIV in clinics in Rio de Janeiro, Brazil: a stepped wedge, cluster-randomised trial. *Lancet Infect Dis* **13**: 10, 852-858.
